# Cure Kinetics Modeling of a High Glass Transition Temperature Epoxy Molding Compound (EMC) Based on Inline Dielectric Analysis

**DOI:** 10.3390/polym13111734

**Published:** 2021-05-26

**Authors:** Erick Franieck, Martin Fleischmann, Ole Hölck, Larysa Kutuzova, Andreas Kandelbauer

**Affiliations:** 1 Automotive Electronics, Engineering Technology Polymer & Packaging, Robert Bosch GmbH, 72770 Reutlingen, Germany; erick.franieck@de.bosch.com (E.F.); martin.fleischmann@de.bosch.com (M.F.); 2 Faculty of Electrical Engineering and Computer Science, Technical University of Berlin, 13355 Berlin, Germany; 3 System Integration and Interconnections Technologies, Fraunhofer IZM, 10623 Berlin, Germany; ole.hoelck@izm.fraunhofer.de; 4 Center for Process Analysis & Technology (PA&T), School of Applied Chemistry, Reutlingen University, Alteburgstrasse 150, 72762 Reutlingen, Germany; larysa.kutuzova@Reutlingen-University.de; 5 Reutlingen Research Institute (RRI), Reutlingen University, Alteburgstrasse 150, 72762 Reutlingen, Germany

**Keywords:** dielectric analysis (DEA), differential scanning calorimetry (DSC), thermomechanical analysis (TMA), kinetics, epoxy molding compound (EMC), inline analytics, process analytics

## Abstract

We report on the cure characterization, based on inline monitoring of the dielectric parameters, of a commercially available epoxy phenol resin molding compound with a high glass transition temperature (>195 °C), which is suitable for the direct packaging of electronic components. The resin was cured under isothermal temperatures close to general process conditions (165–185 °C). The material conversion was determined by measuring the ion viscosity. The change of the ion viscosity as a function of time and temperature was used to characterize the cross-linking behavior, following two separate approaches (model based and isoconversional). The determined kinetic parameters are in good agreement with those reported in the literature for EMCs and lead to accurate cure predictions under process-near conditions. Furthermore, the kinetic models based on dielectric analysis (DEA) were compared with standard offline differential scanning calorimetry (DSC) models, which were based on dynamic measurements. Many of the determined kinetic parameters had similar values for the different approaches. Major deviations were found for the parameters linked to the end of the reaction where vitrification phenomena occur under process-related conditions. The glass transition temperature of the inline molded parts was determined via thermomechanical analysis (TMA) to confirm the vitrification effect. The similarities and differences between the resulting kinetics models of the two different measurement techniques are presented and it is shown how dielectric analysis can be of high relevance for the characterization of the curing reaction under conditions close to series production.

## 1. Introduction

The automotive sector currently experiences a significant transformation incited by trends like autonomous driving, connected vehicles, electrification of the powertrain and shared mobility. It is predicted that these trends will cause major growth in demand of high-performance electronic devices [1]. Moreover, the requirements placed on electronics will also become increasingly demanding. Requiring, for example, their stability at high operating temperatures (>175 °C up to 200 °C) or their ability to withstand long-term exposure to harsh conditions such as hot oils or humidity [2]. For this reason, it is crucial to protect the fragile electronic components via a direct packaging process from the environment while at the same time ensuring good electrical insulation.

Epoxy molding compounds (EMCs) display outstanding chemical resistance, mechanical properties, good adhesion and electrical insulating properties. Therefore, epoxies are frequently used in the electronic and microelectronic industries for packaging of semiconductor devices and microchips and over-molding integrated circuits, hybrid circuits and transistors [3,4,5,6]. Packaging is often done by the transfer molding process, as it has high mass throughput and low tooling costs compared to other packaging alternatives, such as direct injection molding and reaction injection molding [7,8].

In practice, it is difficult to optimize the industrial process and to guarantee high product quality consistently [9]. This is because direct process control during the encapsulation process is difficult to achieve and real-time information on the state of the cross-linking of the material during curing is very difficult to access [10].

To understand the curing progress of the material and its consequences on the final EMC properties, studying the cure kinetics is essential [11]. Typically, this is done offline using methods such as dynamic scanning calorimetry (DSC) in combination with iso-conversional kinetic analysis [12,13,14,15,16,17,18,19,20,21]. DSC has proven to be a powerful tool in the characterization of the curing kinetics of numerous thermosetting materials [11,22,23,24]. Although DSC is very well suited for generating kinetic models, it holds several disadvantages. Firstly, it is limited to off-line analysis in laboratories [25] and cannot be applied as in-process sensor for production control. It is difficult to simulate process conditions such as material preheating and resin flow through a mold cavity. Moreover, the final phase of curing especially may not always be adequately described and predicting technological properties by DSC-based models may lead to faulty conclusions [26].

These drawbacks have given rise to alternative in situ cure monitoring methods based on Raman spectroscopy [27], IR-spectroscopy [26,28,29,30], ultrasonic monitoring [31] or dielectric analysis (DEA) [9,25,27,29,32]. DEA especially holds great potential for industrial application as a process control tool to inline monitor EMC cross-linking in molding tools. It is applicable for measuring opaque materials, which impose particular challenges for optical methods. DEA displays a comparatively robust and simple design with regard to cable routing and sensors, and offers the possibility of fast data processing [33].

Here, we present the inline dielectric process monitoring and kinetic analysis of the curing of a commercially available, high-glass transition temperature (>195 °C) EMC which is suited for the packaging of electronic components. The dielectric measurements were carried out under near-process conditions. The DEA data were evaluated using a model-free (isoconversional) kinetic approach (Friedman method) and a model-based (Kamal-Sourour method) kinetic approach. For comparison, DSC-based kinetic characterization of the EMC was performed as well. The two types of methods are critically discussed. Complementary thermomechanical analysis (TMA) of molded parts was carried out to determine the glass transition temperature and to establish to which extent the calculated kinetic models agree with the temperature-dependent mechanical performance of the materials. We demonstrate how DEA can beneficially be employed to adequately characterize the curing behavior of EMC under near-process conditions.

## 2. Materials and Methods

### 2.1. Materials

**Scheme 1:**
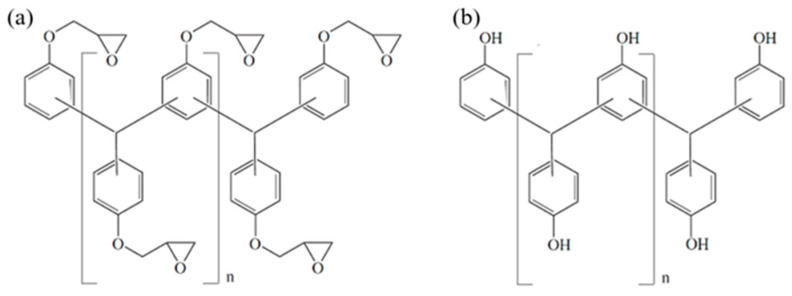
Basic chemical structures of the used multifunctional epoxy resin (**a**) and the multifunctional phenolic hardener (**b**).

A commercially available pre-mixed EMC with a high filler content (83% spherical silica particles) and containing a nucleophilic curing agent was investigated. The basic chemical structure of a multifunctional epoxy resin is given in Scheme 1a and for a multifunctional phenol hardener is given in Scheme 1b. The material was received in pellet form and was of black color. The cured resin possesses a very dense cross-linked network and displays a high *T_g_* of around 195 °C when cured at 175 °C for two minutes and a subsequent post mold cure process at 175 °C for four hours [5,34]. The material was stored at 2 °C and heated to room temperature for >8 h prior to use.

### 2.2. Differential Scanning Calorimetry (DSC)

DSC measurements were performed with a DSC 204F1 Phoenix^®^ (Netzsch Gerätebau GmbH, Selb, Germany) with an integrated auto-sampler. All measurements were conducted under nitrogen atmosphere with a N_2_ flow rate of 40 mL/min. For each measurement, about 20.2 ± 0.6 mg of the pre-mixed resin was weighted into aluminum crucibles (Concavus Pan And Lid From Al, Netzsch Gerätebau GmbH, Selb, Germany), which were sealed and exposed to a temperature ramp ranging from 20 to 220 °C with five heating rates (2, 5, 10, 15 and 20 °C/min). All DSC experiments were repeated three times. The changes in enthalpy were recorded and analyzed using the Proteus Thermal Analysis software (Netzsch Gerätebau GmbH, Selb, Germany). The data were exported to the Kinetics Neo Software (Netzsch -Gerätebau GmbH, Selb, Germany), with which the kinetic parameters for the Friedman (iso-conversional method) and Kamal-Sourour (model fitting method) models were obtained.

The degree of cure (α) directly correlates with the measured heat flow ($\Delta H_{t}$) during the reaction as follows:

(1) $$\alpha_{t}=\frac{\Delta H_{t}}{\Delta H_{Total}}$$

where $\alpha_{t}$ represents the degree of cure at a specific time, $\Delta H_{t}$ is the overall released heat at a specific time and $\Delta H_{Total}$ corresponds to the overall released heat during the complete reaction.

### 2.3. Dielectric Analysis (DEA)

**Scheme 2:**
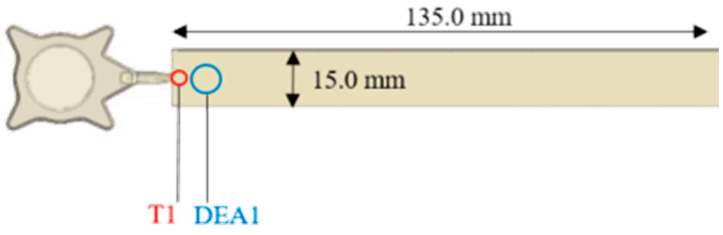
Design and location of the inline sensors in the processing equipment. Thermocouple type K near the gate (T1) and monotrode for dielectric analysis (DEA1).

The dielectric measurements were carried out with a 4/3RC monotrode (Netzsch Gerätebau GmbH, Selb, Germany) and a temperature sensor thermocouple type K (Kistler Instrumente AG, Winterthur, Switzerland), which were connected to a DEA analyzer (DEA 288 Epsilon, Netzsch Gerätebau GmbH, Selb, Germany). The sensors were integrated into a slit-mold-cavity (135.0 × 15.0 × 1.0 mm), that was mounted on a transfer mold press. The DEA and temperature sensors were located at the entry point of the slit-die-cavity. The position at which the DEA sensor was located in the equipment is schematically illustrated in Scheme 2.

The tested EMC was in direct contact with the sensor via a capacitor arrangement. A sinusoidal voltage was applied, and an electric current (2 × 10^−7^ A) was measured as the response. This electric current is caused by the alignment of the dipoles present in the resin in response to the applied external field and depends on the mobility of the involved charge carriers. Changes in electrical current with time indicate changes in ion mobility due to cross-linking of the resin and, thus, represent a measure for network formation [35].

The measurements were started manually, and the data were recorded electronically. The experiments were carried out at two different operational frequencies, 10 and 100 Hz. For kinetic analysis, data from the measurements performed at 10 Hz were used, because of the higher sensitivity towards the end of the reaction. Curing of the EMC was done at five isothermal temperatures (165, 170, 175, 180, 185 °C). The cure time was set to 360 s. Since the time scale of a typical industrial process is, e.g., 90 to 180 s at 175 °C, this cure time was selected to ensure complete curing of the composite and to provide the entire conversion profiles. All isothermal measurements were repeated three times. The evaluation method of the recorded data is presented in the results section.

The response measured using dielectric analysis was the ion viscosity ρ (Ohm cm). This corresponds to the specific resistivity, which is the reciprocal of the specific conductivity σ (S/cm) [33,35]:

(2) $$\rho =\frac{1}{\sigma }$$

The specific conductivity σ can then in turn be expressed by the following equation:

(3) $$\rho =\frac{1}{q\;\mu \;n}$$

where q is the electric charge (coulombs), n is the free ion concentration (cm^−3^) and μ is the free ion mobility (cm^2^/(Vs)) which can be expressed as:

(4) $$\mu =\frac{q\;D}{k\;T}$$

where D is the diffusion coefficient (cm^2^/s), k the Boltzmann’s constant (eV/K) and T is the absolute temperature (K). If the ions present in the thermoset are modeled as spherical particles, the Stokes-Einstein Relation can be used to express D:

(5) $$D=\frac{k\;T}{\left(6\;\pi \;\eta \;r\right)}$$

where η is the mechanical viscosity and r the radius of the ions when modeled as spheres. By combining Equations (3)–(5), we obtain the Equation (6), which illustrates why ion viscosity is an appropriate quantity for monitoring the curing of a thermosetting material:

(6) $$\rho =\frac{6\;\pi \;\eta \;r}{q^{2\;}n}$$

Equation (6) shows that the ion viscosity ρ can be expressed by the mechanical viscosity η and the free ions present in the thermoset. The mechanical viscosity increases during curing, which leads to an increase in the DC resistivity, due to the forming polymer network, that causes a mobility reduction of the free ions [33].

For kinetic analysis, the ion viscosity was converted into the cure index (a), which represents the ratio between the recorded change in signal and the complete signal shift from the minimum to the maximum measured ion viscosity (in log scale) according to Equation (7) [26]:

(7) $$a_{t}=\frac{log\left(\rho_{t}\right)-log\left(\rho_{min}\right)}{log\left(\rho_{\max}\right)-log\left(\rho_{min}\right)}$$

where $log\left(\rho_{t}\right)$ is the decimal logarithm of the measured ion viscosity signal at a given time. The $log\left(\rho_{min}\right)$ corresponds to the minimum ion viscosity. At $log\left(\rho_{min}\right)$, the charge carriers possess the highest mobility. The maximum ion viscosity, expressed as $log\left(\rho_{\max}\right)$, is determined from the maximum height of the ion viscosity profile (when the slope of its first derivative with respect to time approaches zero).

### 2.4. Model-Free (Iso-Conversional) Kinetic Analysis

For kinetic evaluation of the thermal and dielectric data, the model-free (iso-conversional) method proposed by Friedman was applied [36].

Kinetic analysis of the DSC data was based on dynamic measurements using temperature ramps from 25 °C to 220 °C of 2, 5, 10, 15 and 20 °C/min. For calculating the kinetic parameters, the differential form of the kinetic expression (given in Equation (8)) was used:

(8) $$\ln{\left(\frac{d\alpha }{dt}\right)}_{i}=\ln\left[f\left(\alpha \right)A_{\alpha }\right]-\frac{E_{\alpha }}{R\;T_{\alpha .i}}$$

where ${\left(\frac{d\alpha }{dt}\right)}_{i}$ represents the change in conversion, α, over time, t, at a specific heating rate, i a set of selected values of conversion. The values of the apparent activation energy, $E_{\alpha }$, at a specific conversion degree were determined from the slope of the linear plot $\ln{\left(d\alpha /dt\right)}_{i}\;\mathrm{vs}.\;1/T_{\alpha ,i}$ and the values of the pre-exponential factor (A) were determined from the intercept of the same linear dependence. The subscripts (α) and (i) also signifies a specific degree of cure for resin and different heating rates.

Kinetic analysis of the DEA data was based on isothermal measurements at the temperatures 165 °C, 170 °C, 175 °C, 180 °C and 185 °C. For calculating the kinetic parameters, an integral form of the kinetic expression derived from Equation (9) was used:

(9) $$\alpha \left(t\right)=A_{\alpha }\exp\left(\frac{-E_{\alpha }}{R\;T_{i}}\right)t$$

The integral form of the model-free kinetic (MFK) method possesses only an analytical solution for isothermal measurements. The conversion degree is described as a function of time α (t). For estimation of the kinetic parameters, Equation (9) is rearranged after taking the logarithm as follows:

(10) $$\ln t_{\alpha .i}=\ln\left[\frac{\alpha \left(t\right)}{A_{\alpha }}\right]+\frac{E_{\alpha }}{R\;T_{i}}$$

The first term of the right side of Equation (10) and the apparent activation energy can easily be obtained from the linear dependence of $\ln\left(t_{\alpha ,i}\right)\;\mathrm{vs}.\;1/T_{i}$. The term “apparent activation energy” is used since this is an empirical parameter that does not correspond to a specific activation energy of a reaction mechanism, but instead describes the overall activation energy of the sum of all reaction equilibria that are simultaneously involved during the curing process at a respective conversion state [12,37].

The differential model-free kinetic calculations were performed using the Kinetics Neo Software (Netzsch Gerätebau GmbH, Selb, Germany) in case of DSC data. The integral kinetic analysis was done using a Microsoft Excel spreadsheet in the case of the DEA data.

### 2.5. Model-Based Kinetic Analysis

As a second approach for kinetic analysis, a model-based approach using the Kamal-Sourour reaction model was used. The Kamal-Sourour reaction model combines autocatalytic behavior with an *n*-th order reaction model and has already been applied earlier to describe the curing of epoxy resin systems [38,39]. It is based on Equation (11):

(11) $$\frac{d\alpha }{dt}=\left(k_{1}+k_{2}\;\alpha^{m}\right)\;{\left(1-\alpha \right)}^{n}$$

where the first rate constant ($k_{1}$) and the exponent (n) describe the *n*-th order reaction and the second rate constant ($k_{2}$) and the exponent (m) express the autocatalytic contribution of the reaction. Both kinetic constants obey the Arrhenius equation. Kinetic analysis was based on temperature ramps using heating rates of 2, 5, 10, 15 and 20 °C/min. Model fitting was performed by minimizing the difference between the measured and the calculated values using the Kinetics Neo Software (Netzsch Gerätebau GmbH, Selb, Germany).

### 2.6. Thermomechanical Analysis (TMA)

The thermomechanical measurements were performed on the thermomechanical analyzer TMA Q400EM (TA Instruments, Alzenau, Germany) equipped with a MCA70 mechanical accessory used in expansion mode with an expansion probe. The measurements were carried out according to ISO 11359-1 and -2. The samples were first heated up from 25 °C to 260 °C with a heating rate of 5 °C/min. Then the temperature was held at 260 °C for 5 min followed by a cooling step to −40 °C with a cooling rate of 10 °C/min. Finally, the samples were heated up again to 260 °C at 5 °C/min. A preload force of 0.10 N and an applied force of 0.10 N was used. Nitrogen was used as a purge gas with a flow rate of 50 mL/min. The analyzed samples were the same that were produced during the inline DEA experiments. The samples were not post mold cured after the mold process in order to check the *T_g_* after the molding process. The glass transition temperature is considered as the abscissa of the intersection point of the tangents to the two linear portions of the sample length change-temperature curve which delimit the change of the dependence slope.

## 3. Results and Discussion

### 3.1. Off-Line Kinetic Analysis Based on Dynamic DSC

#### 3.1.1. Model-Free Kinetic Analysis

First, DSC-based kinetic characterization of the EMC was performed as a reference method. The kinetic characterization using DSC traces was based on the iso-conversional (model-free) kinetic analysis method proposed by Friedman [36] and the model based Kamal-Sourour approach [38]. The kinetic parameters obtained are compared to literature data for other epoxy phenol systems.

Table 1 summarizes the results from the DSC measurements. 

**Figure 1:**
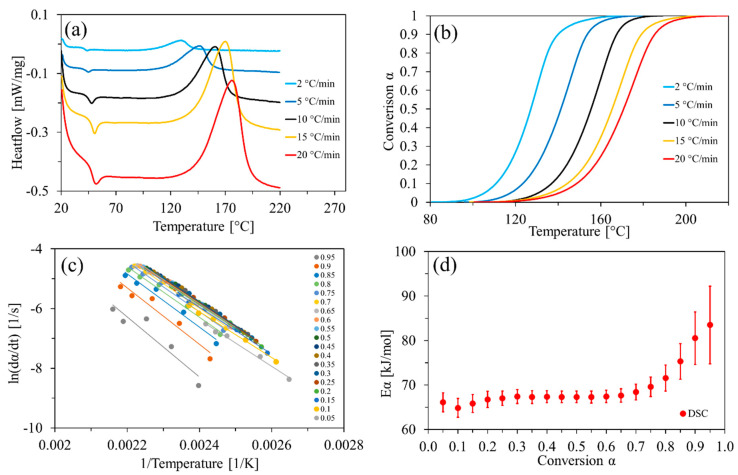
DSC evaluation: (**a**) Heat flow of EMC at different heating rates plotted against the temperature; (**b**) Conversion α plotted against temperature at different heating rates; (**c**) natural logarithm of the reaction rate plotted against the reciprocal of the absolute temperature at specific degrees of cure with the corresponding regression lines (Friedman method); (**d**) apparent activation energy plotted against the degree of cure (Friedman method).

Figure 1 summarizes the kinetic evaluation of EMC curing using dynamic DSC.

For clarity, in Figure 1 only one measurement per heating rate is shown. In each measurement two enthalpy peaks are observed. The first small endothermic peak at around 40–50 °C indicates an enthalpy relaxation of the material. The second larger exothermic peak corresponds to the actual curing reaction. It can be seen that the exothermic peak shifts to higher temperatures with increasing heating rates. This behavior is known for epoxy thermosets [40]. In Figure 1b the calculated values of conversion (according to Equation (1)) are plotted against the corresponding temperatures for the five heating rates. The shift of the reaction onset and end temperatures towards higher temperatures with increasing heating rate can here be observed as well. Furthermore, the slopes of the conversion profiles slightly differ at high conversions (>0.9). At higher heating rates (15 and 20 °C/min) conversion proceeds faster than at low heating rates (2 °C/min) where conversion decelerates earlier. This influence of the heating rate on the curing progression of thermoset materials has been observed before in the literature and can be attributed to the entry of vitrification of the epoxy resins. Vitrification occurs only when the glass transition temperature exceeds the curing temperature and the reaction changes from a kinetically driven reaction to a diffusion-based one, which leads to a slower cure progression. This is usually the case for isothermal curing conditions below the maximum *T_g_* of the cured epoxy resin or for low heating rates (0.2 to 3.0 °C/min), where the *T_g_* of the curing epoxy resin surpasses the temperature profile, due to a faster cure reaction [41,42].

Figure 1c shows the natural logarithm of the reaction rate plotted against the reciprocal of the absolute temperature as calculated by the differential Friedman method (Equation (8)). The data points are classified according to the corresponding conversion values in 0.05 steps.

In Figure 1d, the apparent activation energy is plotted against conversion. At the beginning of the reaction (α < 0.3) *E*(α) increases slightly from 64 to 67 kJ/mol. For α between 0.3 and 0.65 the apparent activation energy is constant and reaches a value around 67 kJ/mol. For α of 0.65 to 0.9 the apparent activation energy increases continuously from 67 to 80 kJ/mol. This tendency is caused by an increase in cross-linking of the thermosetting network that leads to more restricted molecular mobility, i.e., higher energy barriers must be overcome for further progress of the reaction. The increase in E(α) indicates that the reaction is becoming progressively diffusion-controlled. In good agreement with our observation, values for apparent activation energy reported in the literature for epoxy-phenol systems lie well between 50 and 90 kJ/mol [23,39].

**Figure 2:**
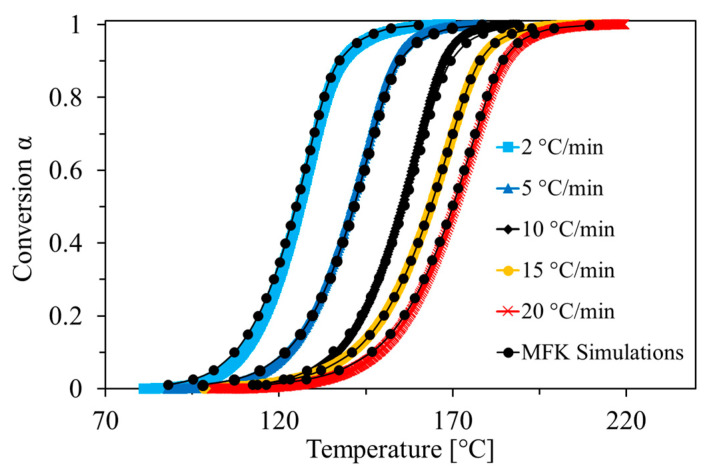
Conversion fits (simulations) after the Friedman method at different heating rates.

In Figure 2, the conversion profiles calculated by the Friedman method are presented (Figure 2, solid lines). They agree well with the experimental values (black dots), demonstrating that the combination DSC/model-free kinetic analysis by the Friedman method can be used to simulate the curing of the EMC with high accuracy.

#### 3.1.2. Model-Based Kinetic Analysis

**Figure 3:**
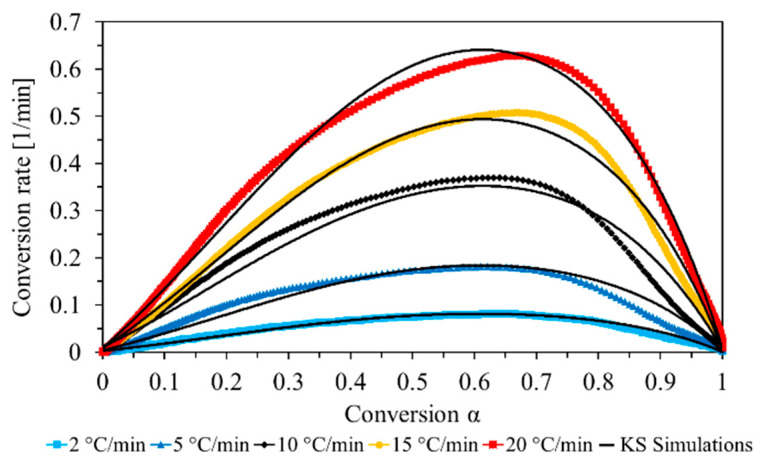
Conversion rate plotted against the conversion α for the experimental data and simulation fits.

The second kinetic method used to evaluate the DSC data was a model-based approach using the Kamal-Sourour reaction model. The visualization of the fitted model is shown in Figure 3 and the values of the kinetic parameters determined by applying Equation (11) are listed in Table 2. The values for the activation energies E1 and E2 (both between 67 and 68 kJ/mol) are very similar to those obtained with the model-free Friedman approach up to 70% conversion (between 64 and 67 kJ/mol). This range of values is also in good agreement with values for *E*(α) found in the literature for the cross-linking of other epoxies (50 to 90 kJ/mol) [11,13,23,39]. The total reaction order found is also in agreement with the literature data with a value of 2.15 (m + n) [43]. The chemical reaction is of the order *n* = 1.06 and for the autocatalytic order, m = 1.09, indicating that both orders contribute similarly to the reaction.

**Figure 4:**
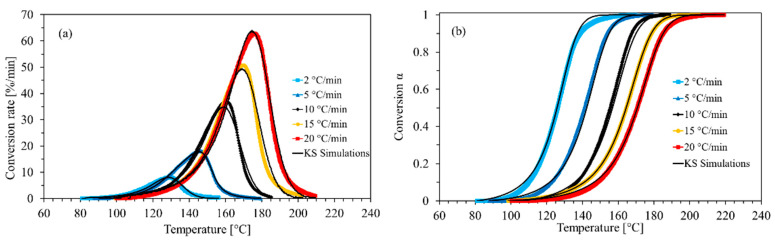
Plots of (**a**) conversion rate fit and (**b**) conversion fit of simulations after the Kamal-Sourour model and measured data at different heating rates.

The simulated conversion rates and conversions based on the Kamal-Sourour model together with the data from the actual measurements are shown in Figure 4. The model simulation, in principle, correlates very well with the experimental data for all temperature profiles. The best matches are found for the high heating rates (15 and 20 °C/min) where the EMC is heated up rapidly. For the lower heating rates (2, 5 and 10 °C/min) the simulation fits are still good, but they do not perfectly coincide with the measured data. This is especially visible in Figure 4b for the heating rate of 2 °C/min, where at the beginning (α < 0.1) and at the end (α > 0.9) the simulation predicts a faster reaction than is observed in the measurement. The minor deviation at the end can be attributed to the effect of entering the diffusion-controlled regime. This means that the Kamal-Sourour model may not describe the diffusion-controlled regime with sufficient accuracy without the implementation of an additional diffusion factor [24,41,42]. Hence, compared to the model-free approach, the predictions obtained by the model-based approach seems to suffer in precision. This will be verified when later in this work, the two DSC based kinetic models will be compared (see Section 3.4, Figure 9b).

### 3.2. Inline Kinetic Analysis Based on Isothermal DEA

#### 3.2.1. Model-Free Kinetic Analysis

Dielectric analysis (DEA) is a non-destructive, electrical measurement technique, which has already been used to monitor progression of cure of EMCs and other resins [44,45,46,47]. In this work, changes in ion mobility are monitored over time as a measure for the crosslinking of the thermoset material. The mobility of the charge carriers depends directly on the resin cure state: in an uncured resin they may move freely and a high ion conductivity is measured. The opposite is true when the resin has cured and a dense cross-linked network has formed [33].

**Figure 5:**
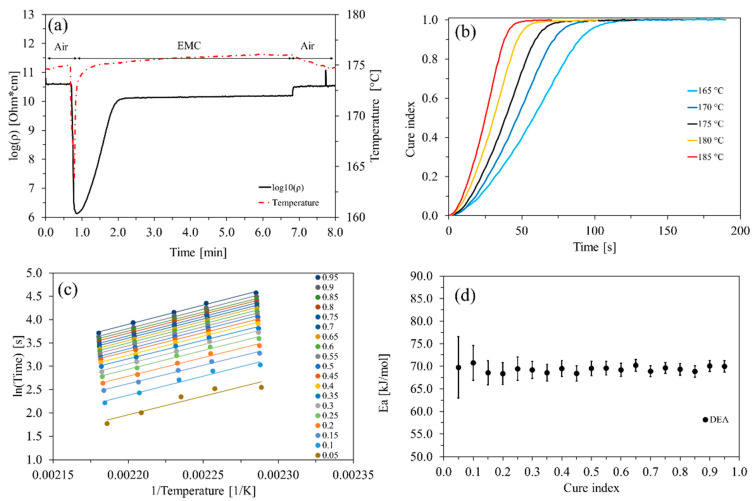
DEA evaluation: (**a**) ion viscosity and temperature plotted against time (**b**) Cure index at different isothermal temperatures plotted against time; (**c**) natural logarithm of time plotted against the reciprocal of the absolute temperature (integral isoconversional method) at specific cure indeces with the corresponding regression lines; (**d**) apparent activation energy E_a_ plotted against the cure index with standard error.

Figure 5a shows a typical ion viscosity curve during the transfer molding process.

The material was transferred through the cavity into the closed tool by a moving plunger. During this time (<1 min) the material was not yet in contact with the sensor. This is seen by the initially constant ion viscosity and the temperature values in Figure 5a. At ~0.7 min, the inflowing material reaches the sensor and an abrupt drop in temperature and ion viscosity is observed. The temperature rapidly stabilizes again at the isothermal temperature as the reaction mass is brought into thermal equilibrium with its surroundings. The ion viscosity increases as curing of the resin proceeds. At the end of the curing process, the ion viscosity signal reaches a plateau level indicating that the cross-linking reaction has ended.

From the DEA profiles, the cure indices were calculated using Equation (7). In Figure 5b the cure indices at the different molding temperatures are plotted against time. Figure 5b shows that at higher curing temperatures, the plateau in ion viscosity is reached faster, indicating that curing proceeds more rapidly with increasing temperature. Interestingly, all curing profiles display a steep gradient even at a curing index as high as 0.9 and above. This only changes at curing indices higher than 0.95, where the slope abruptly decreases and rapidly approaches zero. This means that the cross-linking reaction still proceeds very fast even at rather high cross-linking degrees towards the end of the reaction. No continuous deceleration of cure seems to take place as far as the ion viscosities are concerned.

The DEA data were further analyzed using the model-free iso-conversional Friedman approach. The Friedman method, like any other differential MFK approach, is especially advantageous for the kinetic evaluation of data obtained with differential measurement methods such as DSC, as the calculations do not result in noise amplification. However, when working with integral data such as those provided by DEA or TGA, using differential approaches like Friedman’s may produce erroneous results. This problem was avoided by using an integral form of Equation (10) instead [12].

The apparent activation energy and the pre-exponential factor were determined by applying the integral isoconversional method for isothermal conditions given in Equation (10). In Figure 5c the natural logarithm of time for the respective cure indeces is plotted versus the reciprocal of the absolute temperature. Linear regression fits the data well. The slopes of the regression lines correspond to the apparent activation energies and their intercepts yield the pre-exponential factors. The fitted regression lines are all parallel indicating only one value for the apparent activation energy, i.e., a constant E_a_ that the activation energy does not depend on the degree of conversion is also observed in Figure 5d. The values for E_a_ remain stable around 69.3 ± 0.7 kJ/mol, which is within the range of the values reported for epoxies in the literature (50 to 90 kJ/mol) [23,39,48].

**Figure 6:**
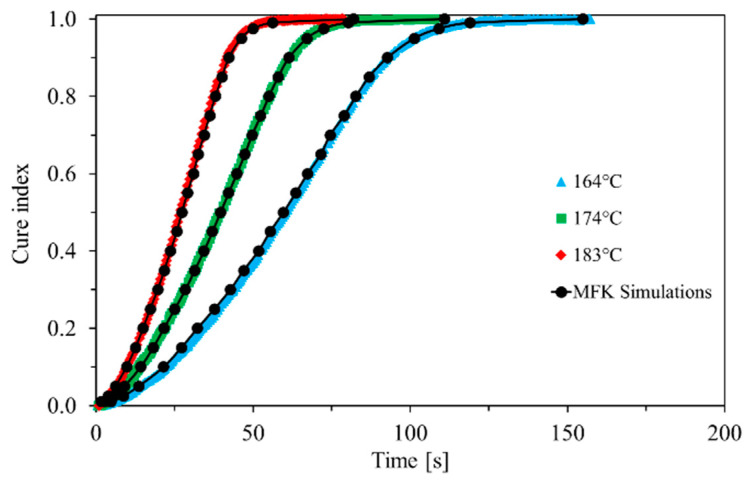
Comparison of isothermal time courses based on iso-conversional calculations (interconnected black dots) and experimental data (red, green and blue dots).

To show the validity of the model parameters (Figure 6) three additional isothermal measurements at 164 °C, 174 °C and 183 °C that had not been used for the model building are compared with the respective MFK simulations by plotting the cure index versus time. The agreement between the measured data and the iso-conversional simulations is very well. This demonstrates that an accurate kinetic characterization of the cross-linking reaction can be achieved by applying the integral iso-conversional method to the real-time DEA-data recorded inline during the curing of EMC within the transfer molding process.

#### 3.2.2. Model-Based Kinetic Analysis

The DEA data were also analyzed using the model-based approach by Kamal-Sourour. Table 3 summarizes the kinetic parameters determined with the Kamal-Sourour model from the isothermal DEA measurements.

The values for the activation energies E1 and E2 are both 69 kJ/mol. This result matches very well the values for the apparent activation energies derived from the isoconversional method of E_a_ = 69.3 kJ/mol. The values of m = 1.07 and *n* = 0.74 indicate a stronger autocatalytic contribution within this model. This seems reasonable since every etherification reaction produces a reactive secondary alcohol as a product and because of the large amount of functional groups present in the multifunctional resin system there should always be a nearby partner allowing the reaction to continue without any delay due to diffusion. Furthermore, the kinetic parameters determined with the Kamal-Sourour model are well within the range of the values reported in the literature (55–90 kJ/mol) [23,39,43,48].

**Figure 7:**
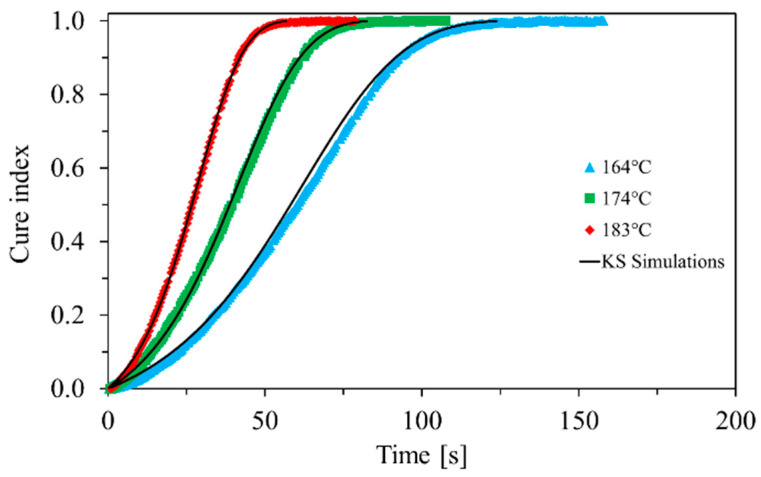
Comparison of Kamal-Sourour simulations (black lines) and measured data (red, green and blue dots).

For validation of the model, three simulation curves derived from the Kamal-Sourour model are displayed in Figure 7 together with the corresponding experimental data. There is good fit between the simulated and experimental data. This demonstrates that an accurate kinetic characterization of the cross-linking reaction can also be achieved by applying the model-fitting approach on inline recorded DEA data within the transfer molding process.

### 3.3. Glass Transition Temperature after the Molding Process

**Figure 8:**
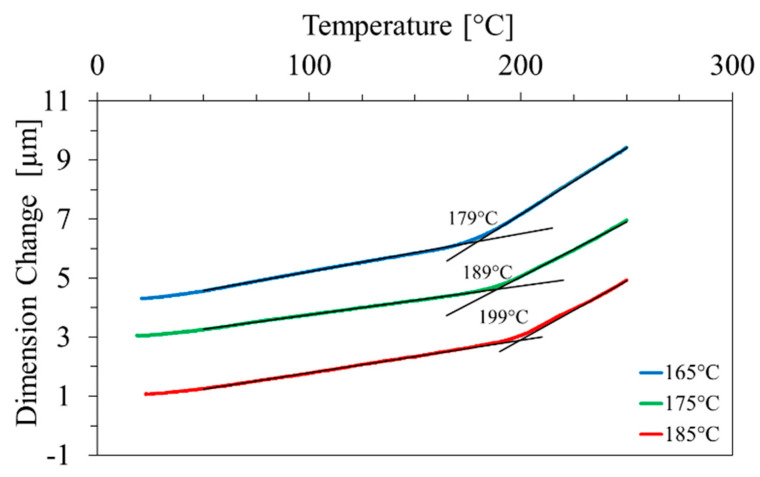
TMA graph of isothermal cured samples, at 165 (blue), 175 (green) and 185 °C (red) for 360 s; *T_g_* is determined by intersection of two tangents on TMA curves and indicated for each measurement (curves are artificially shifted along Y axis for better visibility).

In the previous sections it was shown that the cure progression can be described using inline DEA based kinetic models, where the kinetic parameters agreed well with the values reported in the literature. However, there remains an open question of whether the material has achieved full conversion at all cure temperatures inside the mold as indicated by the cure index. To address this, TMA investigations were carried out with the cured specimens to determine the *T_g_*, since it correlates with the extent of cross-linking. The results are presented in Figure 8.

Depending on the curing temperature, the thermal expansion shows some interesting features. The sample cured at 165 °C exhibits the lowest *T_g_* at 179 °C. The sample molded at 175 °C shows *T_g_* around 189 °C, while the sample molded at 185 °C shows the highest value for *T_g_* at 199 °C. The multi-functional EMC reaches higher *T_g_* with increasing mold temperature. This behavior can be explained very well by the material undergoing vitrification and therefore entering a diffusion-controlled regime during the isothermal curing process inside the mold. This means that the materials cured at higher temperatures have reached a higher degree of conversion and, consequently, a higher cross-linking degree.

Similar observations were found by other groups as well. Typically, this dependence of the *T_g_* on the mold temperature was attributed to the *T_g_* surpassing the curing temperature and thus leading to vitrification of the thermosetting network during the cross-linking reaction, causing the reaction progress to stagnate due to the very slow reaction rate [24,49,50,51].

It is thus concluded that the sudden end of the curing reaction as indicated by the clear flattening of the cure index profiles (Figure 5b) reflects solidification of the resin and transition to the diffusion-controlled reaction regime. The cure index at high conversion degrees indicates when the reaction effectively comes to a halt because of vitrification of the resin. Thus, the kinetic characterization of the EMC based on the inline DEA data does not actually describe when the material reaches full conversion, but rather when the reaction effectively becomes diffusion-controlled inside the mold. This is an important information since it allows identifying the time point when the mold may be opened to remove the processed good.

### 3.4. Comparison of DEA and DSC Kinetic Models

**Figure 9:**
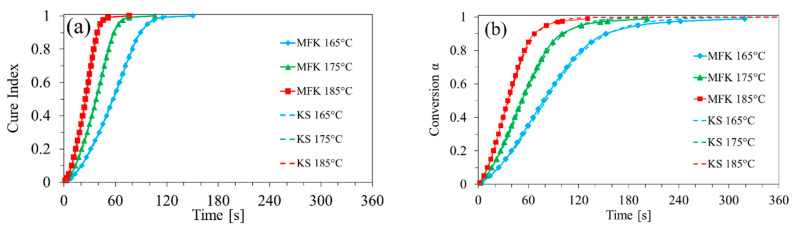
Cure simulations based on DEA (**a**) and DSC (**b**) with model free kinetics (MFK) and the Kamal-Sourour model fitting (KS) for a time period of six minutes corresponding to the cure time inside the mold tool.

In the following section the kinetic models derived from DSC and DEA are compared in more detail. Figure 9 shows simulated isothermal curing profiles of the EMC based on DSC and DEA measurements.

Figure 9 depicts the cure index (DEA, Figure 9a) and the conversion degree (DSC, Figure 9b) versus curing time of the molded samples. In the Figure, the profiles obtained with the model free and the model based kinetic analyses are superimposed for the DEA (in Figure 9a) and the DSC (in Figure 9b) data. The good agreement between both models is consistent with the similarity of the determined kinetic parameters for both kinetic analysis methods (Table 1 and Table 2). This illustrates that both the model-free and the model-based mathematical approaches can equally well be used to describe and quantitatively model the kinetic profile of the curing EMC for both DSC and DEA measurements.

However, the predictions of the DSC-based models are very much different from the predictions of the DEA-based models. This difference is evident when comparing the time scales of the calculated curing profiles for the two experimental methods used to provide the raw data basis for the kinetic analysis.

The conversion profiles derived from DEA data come almost to an abrupt stop towards the end of the reaction. Based on the *T_g_* measurements presented in Section 3.3, it was concluded that this is due to entering the diffusion-controlled regime. Hence, at this stage a conversion degree of 100% in terms of complete functional group transformation is not yet necessarily attained. In contrast, curing isotherms calculated from dynamic DSC data predict much longer reaction times with lower reaction rates at conversions >0.9. The calculated isotherms based on DEA predict the end of cure in approximately half of the time than the DSC simulations. For instance, DEA predicts the end of cure in 75 s at 185 °C, 105 s at 175 °C and 150 s at 165 °C, while with model parameters of DSC the simulation predicts an end of cure in 140 s at 185 °C, 210 s at 175 °C and 350 s at 165 °C, respectively.

Since the calculation of the DEA cure index always refers to the change of the total signal at the specified temperature, the cure index is only a relative value. It depends on the isothermal temperature applied during molding. DSC-based models, in contrast, are based on the conversion α which is derived from the total reaction enthalpy. One of the prerequisites for successful calculation of activation energies by MFK is that the enthalpy integrals determined via DSC do not depend on the used heating rate [12], i.e., the total reaction enthalpy has always the same value independent of the applied temperature profile. Hence, DSC assumes full conversion and α reflects an absolute estimation of the conversion degree in contrast to DEA.

The DSC simulations, therefore, predict that the material reaches full conversion within the timeframe of 360 s for all isothermal temperatures (165–185 °C) (Figure 9b). This implies that the material should reach its maximum *T_g_*-value independent of the temperature at which curing is performed. This, however, is not in accordance with the *T_g_*-values that were actually measured by TMA. The *T_g_*s were in the range from 179 to 199 °C (Figure 8). Since TMA experiments reveal that *T_g_* and the achieved curing states are different and dependent on the applied isothermal temperatures, it would have been expected that the isothermal DSC simulations predict different final curing stages as well. However, this is not the case according to Figure 9b.

While DSC predicts systematically higher conversion degrees (up to full conversion) than experimentally observed based on the *T_g_* of the molded material, DEA data suggest that conversion is strongly dependent on the applied isothermal curing temperature and yields typically lower values than 100% conversion of the material.

**Figure 10:**
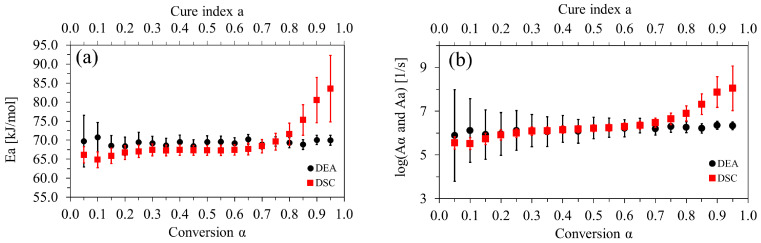
DEA and DSC: Comparison of the apparent activation energies (**a**) and apparent pre-exponential factors (**b**) determined via isoconversional methods of DEA (black dots) and DSC (red squares) with the corresponding standard errors.

This interpretation is also supported by the conversion dependent activation energy profiles depicted in Figure 10.

Figure 10 shows the apparent activation energies (Figure 10a) and pre-exponential factors (Figure 10b) obtained for the DEA- and DSC-based iso-conversional analyses against the conversion α for DSC (Figure 10a red squares) and against the cure index a for DEA (Figure 10a black circles), respectively. The pre-exponential factors obtained from the two measurement techniques follow a similar trend as the apparent activation energies. Therefore, only the differences in the apparent activation energies will be briefly discussed.

The apparent activation energies show comparable values between 67 and 69 kJ/mol for a wide range of conversion degrees and cure indices (α or a between 0.2 and 0.8). At α or a below 0.2, the apparent activation energy determined by dynamic DSC is about 5 kJ/mol lower than that calculated from isothermal DEA. However, this difference is not statistically significant. In contrast, for α values above 0.8, the apparent activation energy determined from DSC data is significantly higher than the apparent activation energy values obtained from DEA measurements.

This difference can be explained by the different experimental conditions used when conducting the DSC and DEA measurements: isothermal (DEA) versus dynamic (DSC) temperature profile. In the diffusion-controlled regime, the material experiencing a dynamic temperature profile up to 220 °C (as applied in the DSC measurements) will behave different from the material being cured under isothermal conditions at a maximum temperature of 185 °C (as in the DEA measurements). Under isothermal cure conditions, the reaction rate will drop rapidly as soon as the *T_g_* of the material exceeds the curing temperature. This will result in an abrupt interruption of the curing reaction (as visible in the steep increase in ion viscosity, Figure 5a). This agrees well with the DEA and the TMA measurements. In contrast, with the non-isothermal DSC measurements, practically complete conversion can be achieved due to the higher temperatures applied. For instance, at the high heating rates of 15 and 20 °C/min, the diffusion-controlled regime is not entered, since the current reaction temperature should always be higher than the current glass transition temperature of the cross-linking network. For the low heating rates, such as 2 and 5 °C/min, this is different. At some point, the diffusion-controlled regime is near since the glass transition temperature approximates the ramp temperature. However, since the temperature is still increasing linearly this allows the cure and therefore the *T_g_* to slowly progress. This means that under these conditions, the EMC does not really vitrify, and a stagnation of the curing by entering the diffusion-controlled regime is avoided. Thereby, the DSC-based kinetic model allows to describe curing without being impeded by the vitrification effect. Similar conclusions have been presented by Granado et al. [23] and other groups [41], where they reported that the temperature profile (isothermal or non-isothermal) had an impact on the entry of the diffusion-controlled regime and therefore on the resulting kinetic parameters for the cure characterization of epoxy-phenol resins.

This means that while dynamic DSC investigates curing under idealized conditions, practically levelling out the contribution of the diffusion-controlled regime to a great extent, isothermal inline DEA includes the phenomena associated with vitrification. Hence, the (dynamic) DSC and (isothermal) DEA kinetic models describe different aspects of the curing behavior of the EMC [42].

Isothermal DEA allows simulation of a more realistic model of curing under close-to-real-production conditions. The presented DEA-based kinetic models can be used to determine the end of the kinetic regime of the reaction at the specific curing temperature and the beginning of the diffusion-controlled regime. As a consequence, in the case of high *T_g_*, EMC does not reach full 100% conversion during the transfer molding process, but rather a “technical” final conversion state that is dependent of the molding temperature. The cure index gives the technically relevant information when the curing in the mold should be interrupted because any further curing will only progress very slowly and can be performed in a separate post-curing phase without occupying the manufacturing tool. Thereby the curing characterization based on DEA describes when a technically sufficient level of conversion has been achieved.

The dynamic DSC model depicts the curing up to a maximum theoretical conversion that can be achieved by higher temperatures with little or no influence of the diffusion-controlled regime. The DEA model describes the curing progress within the process up to the stop of the kinetic reaction due to the material vitrification, as the glass transition temperature surpasses the curing temperature.

This is also reflected by the kinetic constants obtained with the model-based analysis of the kinetic data that are collected in Table 4.

The kinetic parameters derived using the Kamal-Sourour model (Table 4) are very similar for the DEA and DSC measurements. The calculated activation energies E1 and E2 correlate very well with a difference of less than 2 kJ/mol. The pre-exponential factors A1 and A2 are also in good agreement for DEA and DSC and seem to have a similar contribution in each model. The order of the autocatalytic reaction m is very similar for both models (DEA: 1.07; DSC: 1.09).

The only notable difference between the DSC-based and the DEA-based models is observed for the reaction order n. With the DEA-based model, a value of *n* = 0.74 was determined, whereas for the DSC-data the reaction order was *n* = 1.06. This difference suggests a higher contribution of the n-th order exponent in the DSC-derived model. This is in agreement with prolonged cure at higher conversion states (α > 0.9) and the EMC achieving higher conversion degrees when subjected to the dynamic DSC measurements. In contrast, the smaller nth order contribution in the kinetic expression based on the DEA data reflects the rapidly dominating restrictions in mobility that the resin sample experiences during isothermal curing leading to lower overall absolute degrees of conversion. In the present study, different sensors were applied to an EMC.

A detailed investigation of the vitrification behavior using DSC was not the aim of this study. Due to the method used here to investigate the highly filled material (silica content > 80%), the sensitivity of the DSC signals was not sufficient for this purpose because of the very low proportions of resin at slow heating. A detailed comparative kinetic study of different types of EMCs using thermoanalytical methods is currently underway and will be reported at a later stage.

## 4. Conclusions

The curing kinetics of a fast curing, high *T_g_*, highly filled, multifunctional epoxy phenol molding compound was analyzed by isothermal inline DEA and dynamic off-line DSC measurements. The determined kinetic parameters for both DEA- and DSC-based models were in range with values from the literature for similar epoxy phenol systems. However, the models derived from the isothermal DEA data differ significantly from the models based on dynamic DSC measurements, especially at high conversion degrees. Due to the used dynamic temperature profiles, DSC describes complete curing under idealized conditions, practically with no vitrification-solidification effects. In contrast, DEA describes the curing process under more realistic and near-production process conditions. We have shown that the DEA profiles also accounts for the solidification of the reaction mixture. Thus, the degree of cross-linking achievable in the material at a given curing temperature becomes dependent on the isothermal curing temperature used and is always less than the value for complete curing as determined by DSC. It reflects the curing degrees that can be achieved practically under near-production process conditions. Isothermal inline DEA is therefore well suited as a laboratory method to analyze and mathematically model the curing of thermosetting reaction masses. Suitable temperature/time profiles for industrial processing (process design) can be defined. Since it can also be applied inline directly in the industrial machinery, it is also a highly versatile process analytical tool to follow the curing process of the reaction mass in molds in-situ as a means for real time process control.

## Figures and Tables

**Table 1:** Exothermic effect expressed as the area of exothermic peak from DSC experiments at different heating rates including the repetitions.

Heating Rate β [°C/min]	Sample Amount [mg]	Peak Area [J/g]
2.0	19.93	24.678
2.0	19.93	25.664
2.0	19.21	26.968
5.0	20.3	27.535
5.0	19.47	26.784
5.0	19.66	26.38
10.0	20.58	28.572
10.0	21.84	29.739
15.0	19.99	34.269
15.0	20.24	34.332
15.0	20.46	35.084
20.0	19.9	33.721
20.0	20.66	35.839
20.0	20.31	32.84

**Table 2:** Kinetic parameters determined using the Kamal-Sourour model based on DSC data.

Kinetic Parameters	Values
E1 [kJ/mol]	67.91
log (A1)	5.65
E2 [kJ/mol]	67.34
log (A2)	6.48
m	1.09
n	1.06

**Table 3:** Kinetic parameters determined using the Kamal-Sourour model based on isothermal DEA data.

Kinetic Parameters	Values
E1 [kJ/mol]	69.34
log (A1)	5.83
E2 [kJ/mol]	69.12
log (A2)	6.87
m	1.07
n	0.74

**Table 4:** Comparison of the kinetic parameters determined by the Kamal-Sourour equation (Equation (11)) via DEA and DSC.

Kinetic Parameters	DEA	DSC
E1 [kJ/mol]	69.3	68.0
log (A1)	69.1	67.4
E2 [kJ/mol]	5.83	5.65
log (A2)	6.87	6.48
m	1.07	1.09
n	0.74	1.06

## Data Availability

Data are available upon request from the authors.
